# iSAT: a visual learning analytics tool for instructors

**DOI:** 10.1186/s41039-016-0043-3

**Published:** 2016-09-01

**Authors:** Rwitajit Majumdar, Sridhar Iyer

**Affiliations:** 1 Inter-disciplinary Program in Educational Technology, Indian Institute of Technology Bombay, Powai, Mumbai, 400076 India; 2 Department of Computer Science and Engineering, Indian Institute of Technology Bombay, Powai, Mumbai, 400076 India

**Keywords:** iSAT, Active learning, Peer instruction, Learning analytics, Cohort analysis, Visual analytics, Teacher training

## Abstract

Interactive Stratified Attribute Tracking (iSAT) is a visual analytics tool for cohort analysis. In this paper, we show how instructors can use iSAT to visualize transitions of groups of students during teaching-learning activities. Interactive visual analytics gives the instructor the affordance of understanding the dynamics of the class of students and their activities from the data collected in their own teaching-learning context. We take an example of a peer instruction (PI) activity and describe how iSAT can be used to analyze its clicker responses. During PI, typically instructors only use histograms to visualize the distribution of clicker responses in the pre- and post-discussion phases. We show that the use of iSAT to analyze clicker data in real time to trace transitions of participants’ responses during various voting phases can support them in planning for their post-PI activities. Seven patterns of transitions that emerge are *aligned*, *returns*, *starburst*, *slide*, *attractor*, *switching*, and *void*. We interpret them in the context of the example. Such transition patterns are neither available in multiple histograms of individual voting phase nor generated in real time to be visualized as a flow diagram. We had conducted two workshops to introduce iSAT to the instructors and demonstrated the workflow of using iSAT with their dataset. Here, we report usefulness and usability data collected from those workshops. In conclusion, we highlight the power of iSAT for instructors to do cohort analysis in their teaching-learning practice.

## Introduction

Technology-enabled active learning practices are transforming both in-class and online teaching-learning scenarios. Many of these active learning strategies can benefit from the availability of logged data. Learning analytics on the data logged during such activities can further help instructors to gain insights and reflect on their practice (Duval, 2011).

**Fig. 1:**
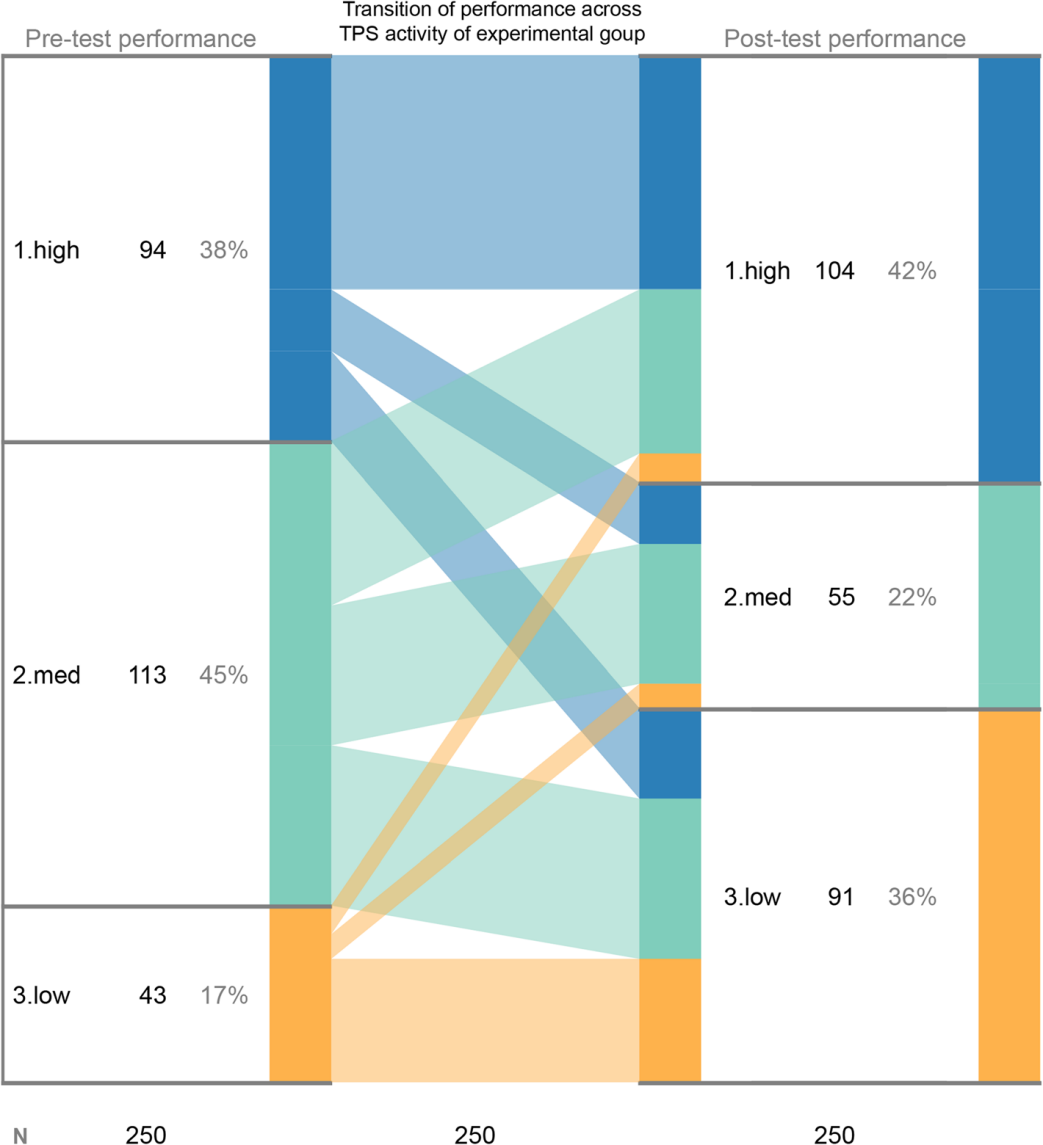
Interactive Stratified Attribute Tracking diagram of performance data of learners

We have developed a tool, Interactive Stratified Attribute Tracking (iSAT), to assist visual learning analytics. The interactive tool helps in analyzing cohorts in educational dataset. For a multi-attribute dataset, we can group data based on criteria set for any attribute value. iSAT can then visualize proportions of these groups and their transitions across attributes. It further assists to interactively analyze patterns that exist in those transitions. Figure 1 shows an example iSAT visualization of cohort transitions between the pre-test scores and the post-test scores of learners. The learners were grouped in cohorts of high, medium, and low achievers based on set criteria of their score. iSAT visualizes proportions of those cohorts and traces their transitions across assessments. This data was reported in a study (Kothiyal et al. 2014), where in a large introductory computer science (CS101) class, learners participated in an active learning activity called Think-Pair-Share (TPS). iSAT helped to answers questions like what proportion of learners possibly benefited from the strategy as indicated by improvement in the post-test score or what proportions’ performance did not change, so possibly, they remained unaffected.

Similarly, users can consider any data attribute collected across a period of time and define cohorts based on stratification criteria on attribute values. iSAT helps to analyze their temporal trends. They can also analyze transitions across attributes, for example, performance and perception, and probe queries like what proportion of high achievers perceive a certain classroom activity as engaging. Such transition patterns remain implicit in the data when stored in a table. Across two attributes, a contingency matrix can capture the transition patterns. But if one needs to track more than two attributes, it becomes difficult to retrieve from a table. iSAT addresses this gap by letting the users to simply upload their dataset, while the tool computes the proportions of the underlying cohorts and visualizes them. This visualization adds value by explicating the transition patterns and highlighting it to visual perception rather than a table matching operation. Further, the tool affords interactive exploration of the transition patterns to find proportions of cohorts in the dataset.

To report patterns in the dataset and get insights in their research context, researchers have previously used the iSAT tool and its analysis method. In this paper, we focus on the instructors and how iSAT can help them to understand the dynamics of their teaching-learning environment and assist in instructional decision-making. In the next section, we introduce a working example of peer instruction (PI), another active learning context, and elaborate how tracing transitions of students’ response through iSAT can help instructors take instructional decisions in that context. We then describe the workflow of how any instructors can use iSAT with their dataset. We report a study of usefulness and usability results of iSAT, done as a part of two introductory workshops on iSAT for instructors. At the end, we summarize the work done and its limitation and indicate future directions of iSAT research and development.

## Example context: PI

**Fig. 2:**
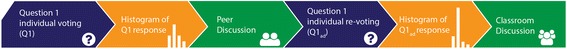
Activity flow in a classic peer instruction (PI) activity

PI (Mazur, 1997) is an instructional strategy to facilitate active learning (Crouch & Mazur 2001, Fagen et al. 2002) . In PI (see Fig. 2 for activity flow), students are engaged in active learning by participating in a deep conceptual multiple-choice question (Q1) on any topic. In the first round, they vote individually (response to Q1). This is followed by a discussion with the neighbor to justify their answer. Then, they re-vote their choice on the basis of the discussion (response to Q1_ad_). For closure, there is an instructor-led classroom discussion regarding the correct answer or clarifying alternate conceptions based on which the wrong options were designed.

Smith et al. (2009) further extended the classic versions of PI for an undergraduate genetics course and added an isomorphic question (Q2) as a third phase of voting (see Fig. 2). An isomorphic question is on the similar concept as Q1 but has minor variations. Later, Porter et al. (2011) investigated the effectiveness of PI with isomorphic questions in the domain of computer science (CS) education.

**Fig. 3:**
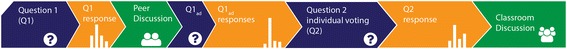
Modified PI activity flow with an isomorphic question (Q2)

Clickers or similar personal response systems are often used as a technology support to collect student responses (Mayer et al. 2009). Instructors may choose to show the variation in distribution of answer choices after first-round voting, after second-round re-voting (stage 2 and stage 5 in Figs. 2 and 3), and after voting in isomorphic question (stage 7 in Fig. 3), in the form of a histogram. Success of the activity is gaged based on shift towards the correct option by majority of the class. Readers interested in implementing PI as an evidence-based instructional practice in different disciplines like physics, chemistry, biology, and computer science can look at the review by Vickrey et al. (2015).

### A reconstructed response dataset of a PI activity

**Fig. 4:**
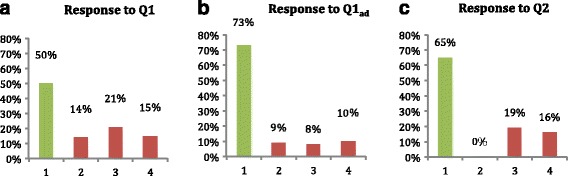
Reconstructed histograms based on accuracy data from Porter et al. (2011). Response distribution of **a** Q1, **b** Q1_ad_, and **c** Q2

Porter et al. (2011) reported a study based on the implementation of PI with an isomorphic question in the Introduction to Computer Architecture (CArch) course. They presented percentage of correct responses for all three phases of voting (Q1, Q1_ad_, and Q2) as a bar chart and also provide the flowchart (tree diagram) of the students’ response accuracy over the isomorphic sequence. We use the reported study just as an illustrative PI activity and to regenerate dataset of responses. We reconstructed random student responses across three PI phases such that it matched their aggregate results. While reconstructing 300 clicker responses as answers to three multiple-choice questions by 100 students, we assumed four options with option 1 as the correct answer for each of the three questions. The incorrect options in each question are linked to an alternate conception in the assumed topic. An example of our generated data is as follows: student #46, response to {Q1, Q1_ad_, and Q2} is {2,1,3}. It means the student was incorrect in the first phase of individual voting, and then, he chose the correct answer during re-voting after peer discussion but was again wrong during voting for Q2. Figure 4 presents the histogram of the voted options that match the study’s overall accuracy values across the different voting phases.

### Limitations of multiple histograms

The response data captured across multiple student voting activities contain details, but its granularity is lost if we consider only aggregate level count and plot it as a histogram for each phase. Such aggregation may limit the understanding of classroom trends by the instructor. Importantly, separate histograms do not convey the pattern of change of students’ response across different activities. The instructors do not know how many students are actually improving after the intervention. It does not highlight portion of the cohort that re-votes an incorrect option or changes from a correct answer to an incorrect one. There is possibility of transition from one wrong option to another one during re-voting. Such cohorts are also missed in the aggregate-level view of the class. For example, from Fig. 4, the instructor does not get to know what proportion of the 21 % of students who voted option 3 are present in the 73 % cohort in the next phase.

Researchers initially were interested to analyze only aggregate-level learning gain during such activities (Hake, 1998). Later, others (Smith et al. 2009; Porter et al. 2011) analyzed transitions of accuracy across attempts by using flowcharts. It is to be noted that the transitions among alternate conceptions, as reflected by responses across the two attempts, are not highlighted. It is interesting to observe those transitions (Wittmann and Black 2014) as they help to decide post-discussion activities (Majumdar and Iyer 2015). To the best of our knowledge, there are no tools available that can assist instructors in tracking the transitions of responses and analyzing patterns in real time. Our earlier paper (Majumdar and Iyer 2015) reported details of various alternatives that researchers had used to analyze PI responses, their limitations with respect to visual analytics, and the advantages of using iSAT. Here, we take the PI context as a working example of instructor’s use of iSAT.

## iSAT to visualize transitions in students’ dataset

**Fig. 5:**
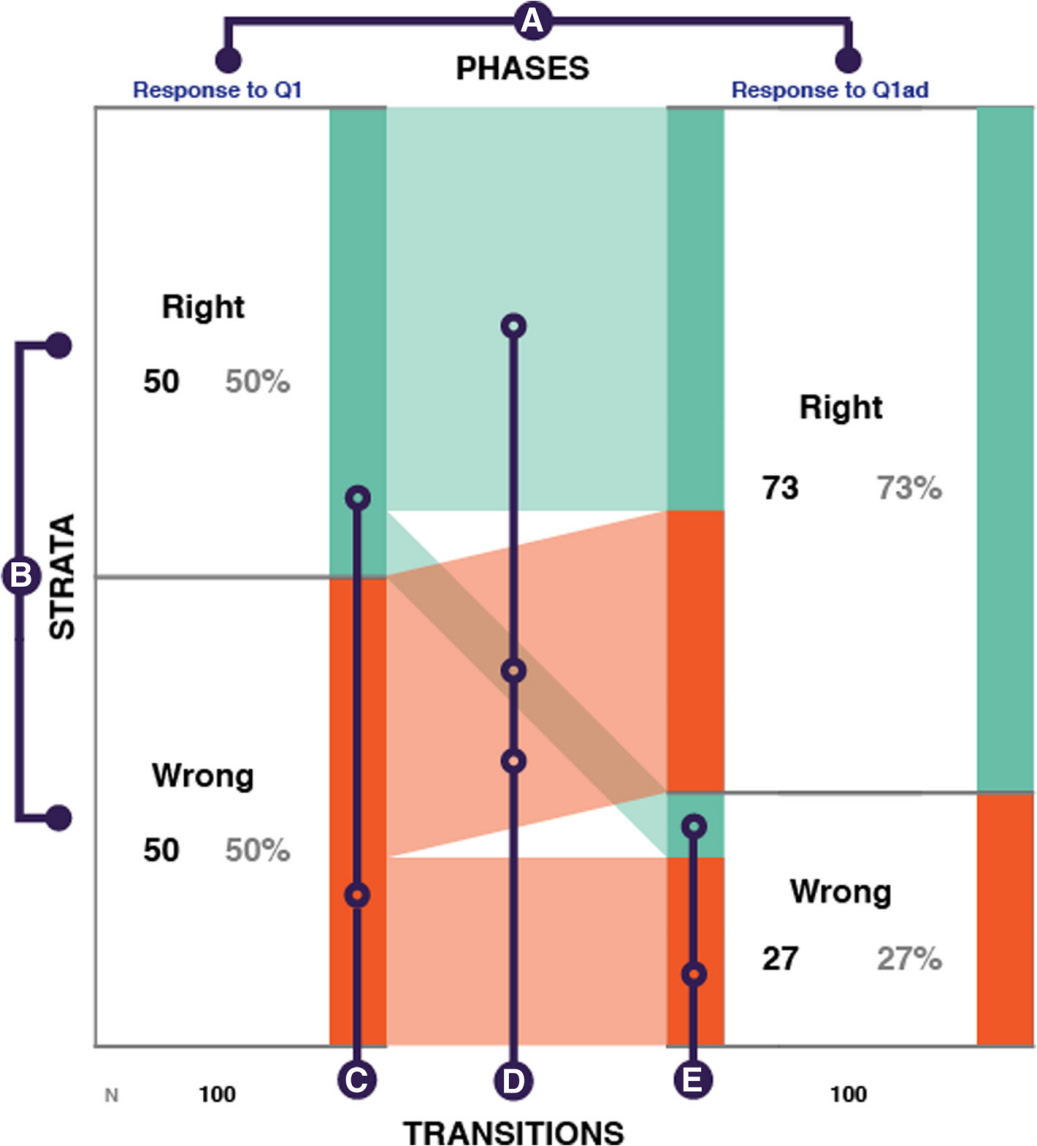
Structure of iSAT. Highlighting A Phases, B Strata, D Transitions. C represents the proportion of the cohort in a phase. E highlights the proportion of the stratum that transits from the previous phase

iSAT diagram is a tool to visualize transition patterns in user datasets. In the context of classical PI, the dataset students’ responses in each phase are the attributes. Figure 5 highlights transitions in accuracy across the two voting phases of the reconstructed PI responses. Each of the initial *response to Q1* and post-discussion *response to Q1*_*ad*_ attempts respectively are represented by a *phase* (see element A in Fig. 5). In each phase, based on attribute values, *strata* represent cohorts. In the case of accuracy, they are “Right” or “Wrong” (see B in Fig. 5). *Transitions* are the links that trace migration of the cohort across phases (D in Fig. 5).

**Fig. 6:**
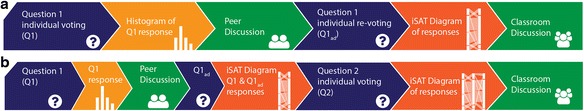
**a** Introducing iSAT in the classical PI activity. **b** Cohort analysis by iSAT for modified PI with isomorphic question

Utilizing iSAT in classical PI (activity flow as shown in Fig. 6a) and one with isomorphic question (activity flow as shown in Fig. 6b) enables an instructor to trace various answering patterns across the voting activities and identify the different cohorts of students based on it and effectively plan the post-PI activities. Thus, iSAT helps to bridge the gap mentioned in the earlier section.

In subsequent sub-sections, we illustrate the detail visual structure of iSAT visualization and the transition patterns it explicates.

### Structure of iSAT visualization

Each phase of iSAT is visualized as a column (see element A in Fig. 5). Each stratum in a phase is represented as a colored bar on the right edge of the column. The height of the bar encodes the proportion of the corresponding stratum in that phase (see element C in Fig. 5). The stacked bars on the left of the column represent the proportion of the stratum that is migrating from the previous phase. For a given stratum, each bar on the left has the representative color of the stratum of the previous phase and height proportional to the number of records that is migrating to the current stratum (see element E in Fig. 5).

Each stratum represents a cohort based on the attribute value in a phase. The links also represent cohorts based on specific transition paths across the phases. For example, the orange upward link (element D in Fig. 5) is the transiting cohort that is incorrect at the first phase and then becomes correct. iSAT helps to visually represent both of these cohorts. The click interaction on both the strata and links provide the corresponding proportion values to the user. This affords the user to interactively explore transition and its proportions.

**Fig. 7:**
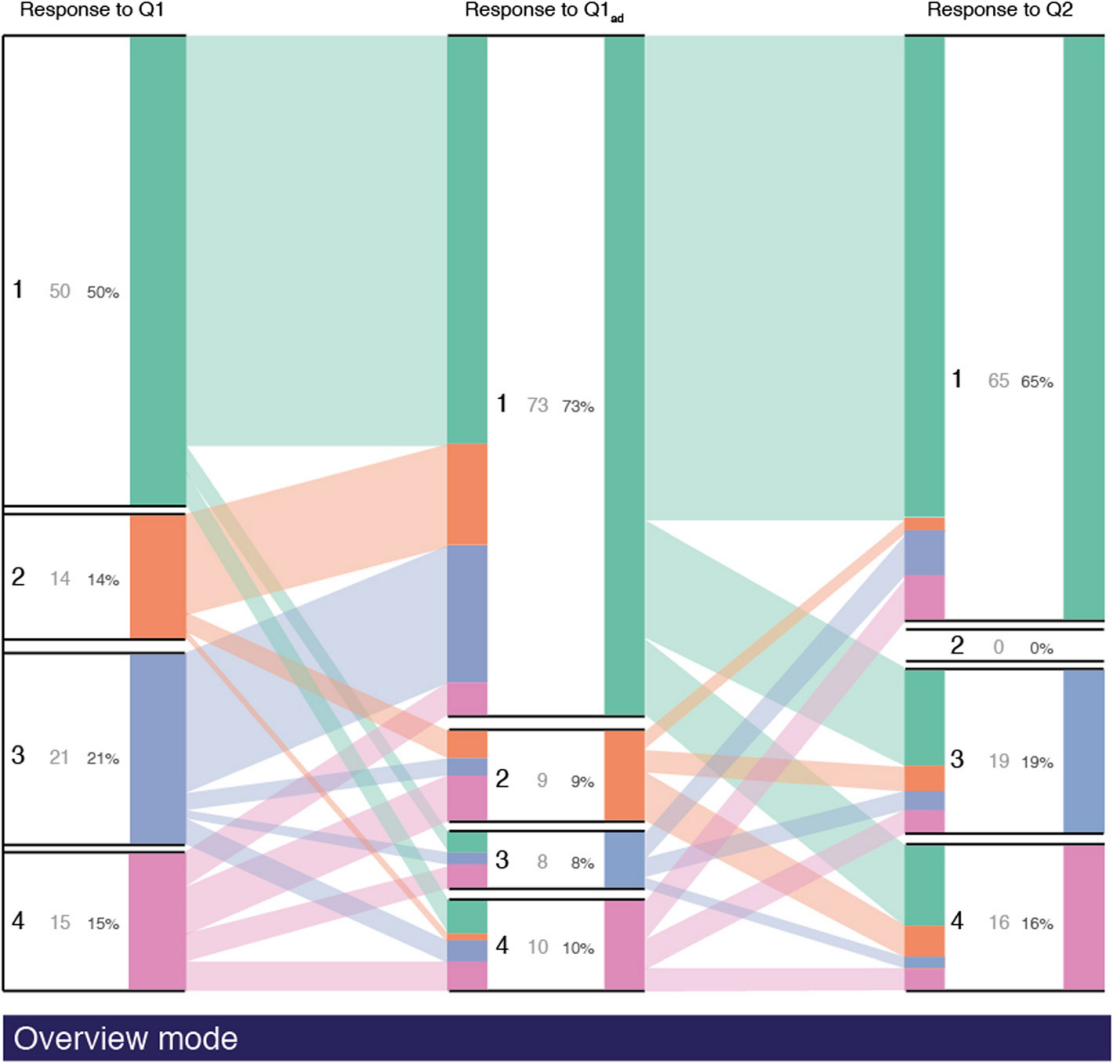
Overview of the transitions in the example dataset

**Fig. 8:**
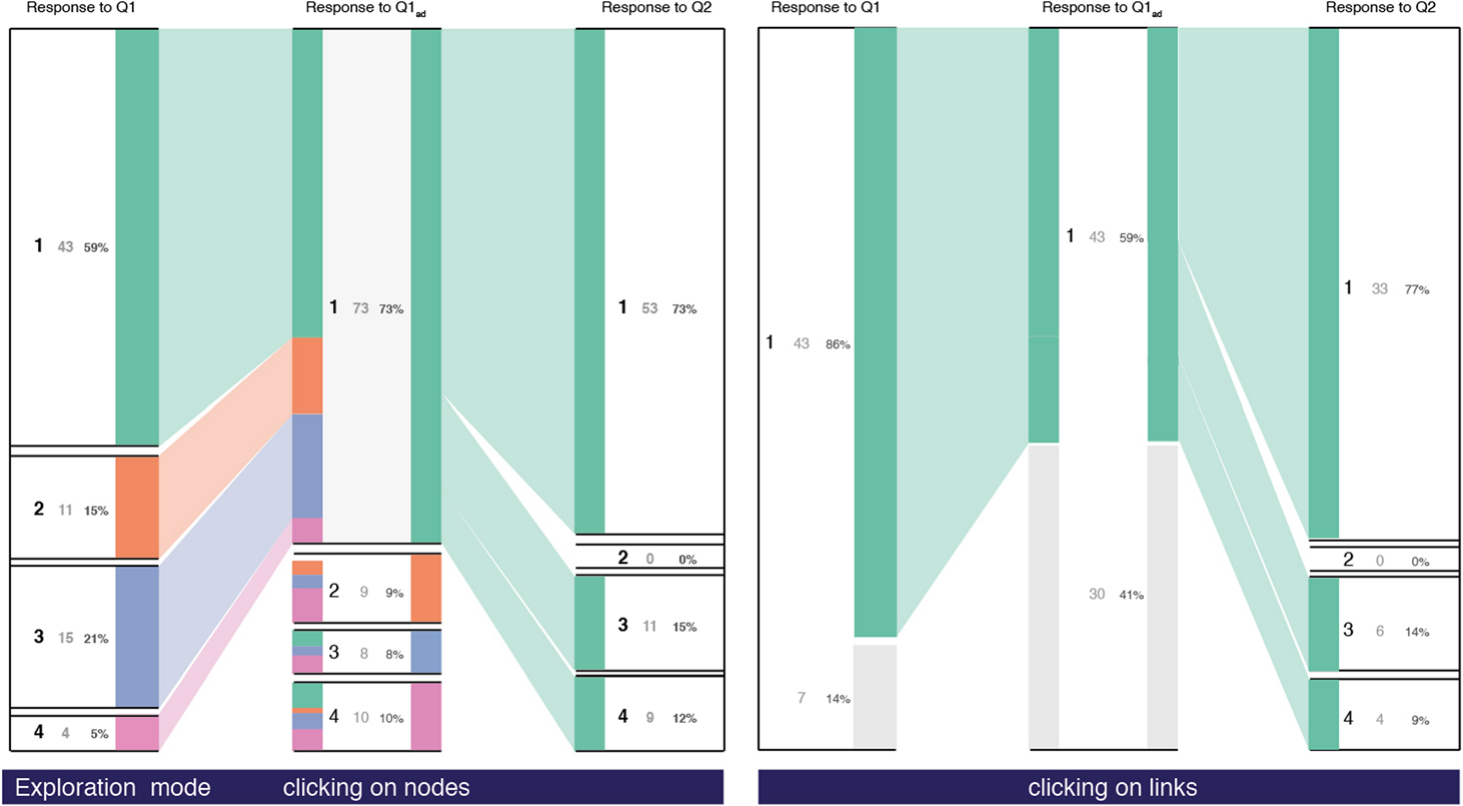
Interaction instances of iSAT for exploring details-on-demand

Figure 6a illustrates the workflow to include iSAT while conducting classical PI. With the data being collected through any clicker system, each collected response data is already linked to a participant by logging the answers corresponding to a device ID. Feeding that clicker data as input, our system computes and visualizes an iSAT diagram. It can help both instructors and researchers to look into a level deeper in the response data than histogram or flowcharts to explicate transition patterns. Such transition patterns can be further categorized to assist instructors to reflect on cohorts in the students and investigate their learning characteristics. Figure 7 shows the iSAT visualization for our regenerated dataset. iSAT has two modes, the Overview mode and the Exploration mode. The Overview mode (see Fig. 7) provides the static distributions of each nodes and transitions in between them. The user enters the Exploration mode once they interact with the visualization through click/tap to explore further details of any cohort represented (see Fig. 8).

#### Overview of PI dataset visualization

The Overview mode provides an overview of the transitions. For example, in Fig. 7, the top block in the second column conveys that 73 % of the students voted option 1 during the re-voting phase. It is seen that the right wall is proportional to 73 % of the full column. The left wall of that block gives distribution of those 73 % cohort with respect to the previous phase. The links (band) on the left and right visualize the cohort that is transiting between any strata of two consecutive phases. The width of the band is proportional to the transitional ratio with respect to the whole population size.

#### Exploration of PI dataset transitions

**Fig. 9:**
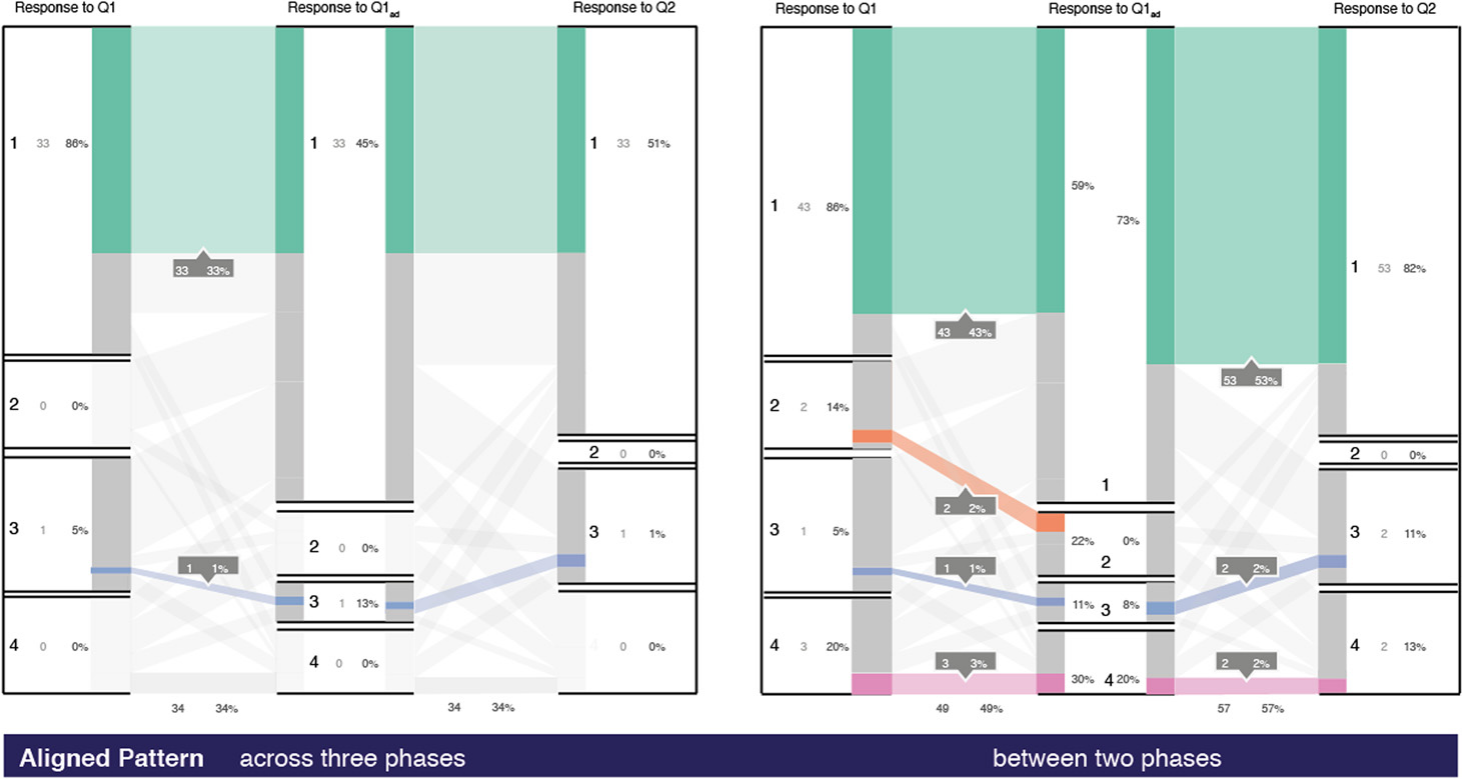
Aligned patterns **a **across three phases and **b** between each two phases

To explore details of proportions, the users can click/tap on either boxes corresponding to the stratum or links corresponding to a specific transition. It helps users to select the cohort whose details need to be explicated. When the cohort corresponds to a specific category in a phase, then there is a transformation of the diagram and in each phase, the distribution of the corresponding cohort is visualized. For example, in Fig. 9a, when the users click on the correct category of response in Q1_ad_ (option 1), they get the details of that 73 % cohort, in which strata and what proportion do they belong in Q1, and their response distribution in Q2. So instructors are informed that 59 % of the people who are correct after re-voting were initially correct and 73 % of them answered the Q2 correctly.

When the user clicks on the band representing a transition cohort, the diagram highlights the migration pattern for that cohort of students across the phases. For example, in Fig. 9b, iSAT visualizes and tracks the cohort that was correct in Q1 and Q1_ad_. Forty-three percent of the population belongs in this cohort. It compromises 86 % of the correct response group during individual voting and 43 % of the correct response during re-voting. Further, it conveys that 77 % of that cohort answers Q2 correctly and in the 23 % of the incorrect responses, 19 %’s answer is option 3.

## Visualizing patterns of transition in iSAT

Based on the transitions that iSAT visualizes, there can be patterns that aggregate transitions of a certain category which can then help interpret the context in which the data is collected. The iSAT tool interactively assists in tracing such patterns across phases and visually representing them.

### Transition patterns in PI dataset and how instructors can interpret them

iSAT explicates the rich transition patterns in the three-phase isomorphic PI activity response. The structure of the visualization gives the instructor an overview of the pattern, and the interactive tool then allows them to explore further details about those transitions. We explored all the 64 possible transition patterns across three voting phases each having four options. Out of them, we defined seven categories of specific transition patterns, which can be interpreted by the instructor who is conducting the PI activity. It also signifies cohorts of interest. Some of the patterns of interest were adopted from the consistency plot analysis done in the context of physics education research to analyze pre-post student responses (Wittmann and Black 2014).

#### Aligned pattern

Aligned cohort remains in the same strata across phases. A bigger population of aligned correct would probably indicate most students had the concept right and hence consistently answered correctly across the voting phases. The instructor can take it as a cue to go forward and introduce the next topic. An aligned cohort to incorrect responses indicates that the cohort requires attention towards learning the concept, as they fail to answer both the isomorphic questions correctly across three phases of voting. For example, in our dataset, 34 % of the population voted the same choice across three phases with 33 % consistently answering the right option and 1 % consistently answering incorrectly without even switching their response from option 3 (see Fig. 9a). If the aligned incorrect population proportion is considerably more, the instructor then knows that the cohort is always stuck in the alternate conception linked to option 3. Further, Fig. 9b shows the alignment between two consecutive phases. Eighty-six percent of students, who gave the correct answer (option 1) in Q1, reaffirmed their choice after the peer discussion. Considering only the first two phases, this cohort represents the *Control Group* (as defined by Porter et al. 2011). Across three phases, they belong to the cohort that contributes to the denominator of the weighted learning gain. Interestingly, pairwise response alignment shows 3 and 2 % of students answer option 4 across the voting phases, respectively, but none consistently get it wrong by answering 4 all the time. Such analysis is not possible by plotting histograms or flowcharts.

#### Return patterns

**Fig. 10:**
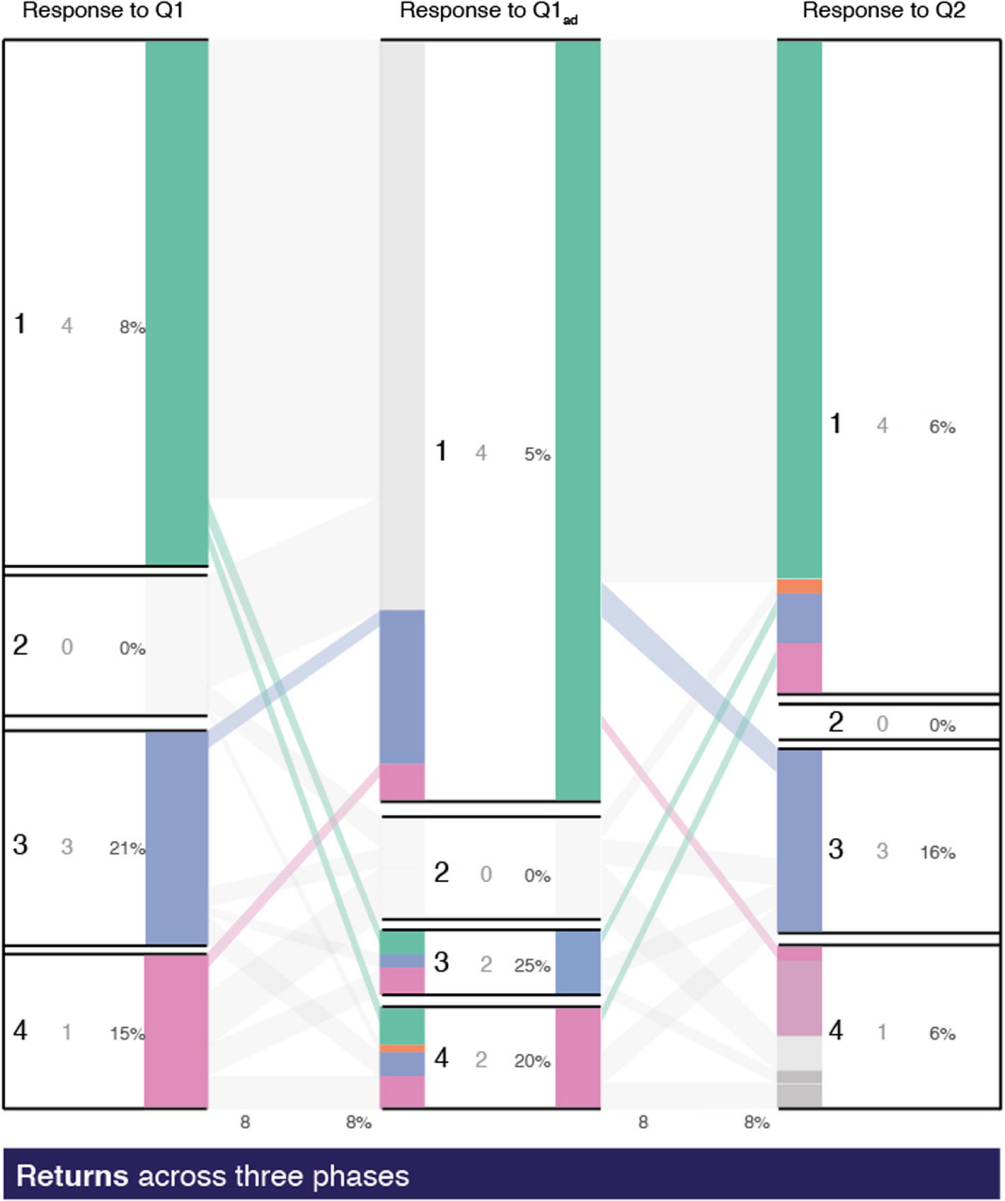
Return patterns across three phases

The portion of the population who gives an initial response and changes it in the second phase but later returns to the original response category forms a return pattern. The instructor can identify the proportion of these students who are inconsistently correct and give specific scaffolds for their learning. In our data, 4 % changed the answer from right to wrong and then again answered Q2 correctly and another 4 % were initially wrong and rectified after PI but answered Q2 incorrectly (see Fig. 10).

#### Starburst

**Fig. 11:**
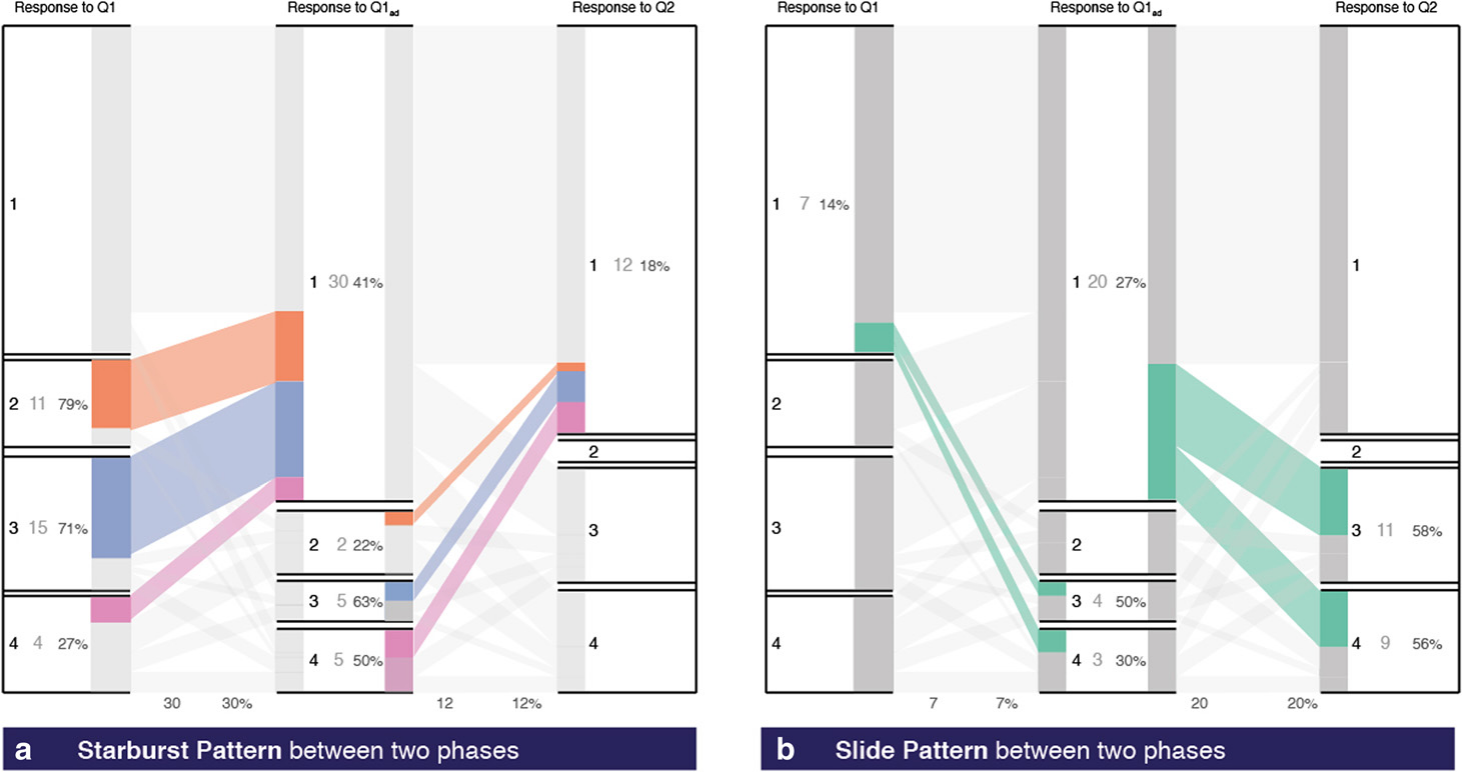
**a** Starburst patterns. **b** Slide patterns

A portion of the cohort that migrates out of a particular stratum in a pre-phase to other more desirable strata in the post-phase generates a starburst pattern. In the context of PI, between two phases of voting, when some students are incorrect in the first phase but go on to become correct in the next phase, they highlight the starburst pattern (see Fig. 11a). In our dataset, 30 % of the students starburst between Q1 and Q1_ad_ and 12 % from Q1_ad_ to Q2. Starburst from the incorrect response is desirable from the first phases. The instructor also identifies the cohort of the *Potential Learner Group* (refer to Porter et al. 2011 for definition) who benefited from the PI activity.

#### Slide

A portion of the cohort that transits from a desirable stratum in the pre-phase to a less favorable stratum in the post-phase generates a slide pattern. In the PI case, the transition from a correct answer to an incorrect answer across two phases of voting generates a slide. Since slide from the correct to incorrect is undesirable, the instructor can create specific activities that address the misconception that may be causing the slide. Figure 11b highlights the cohort that slides from a correct answer to an incorrect response across voting phases in our example. Seven percent of the students slide after individual voting, and 20 % slide during Q2.

#### Attractor

A portion of the cohort that migrates into a particular stratum in a post-phase from other strata in the pre-phase generates an attractor pattern. Considering the answer option 3, the attractor pattern would highlight the cohort that has transitioned from other strata in the pre-phase to the one of option 3 in the post-phase. For only one desired stratum, tracing the attraction pattern corresponding to option 1, we shall get the same plot as starburst. The instructor can compare attractor patterns of the incorrect response options and can correspondingly decide which alternate conception can be addressed in the post-PI discussion.

#### Switching

**Fig. 12:**
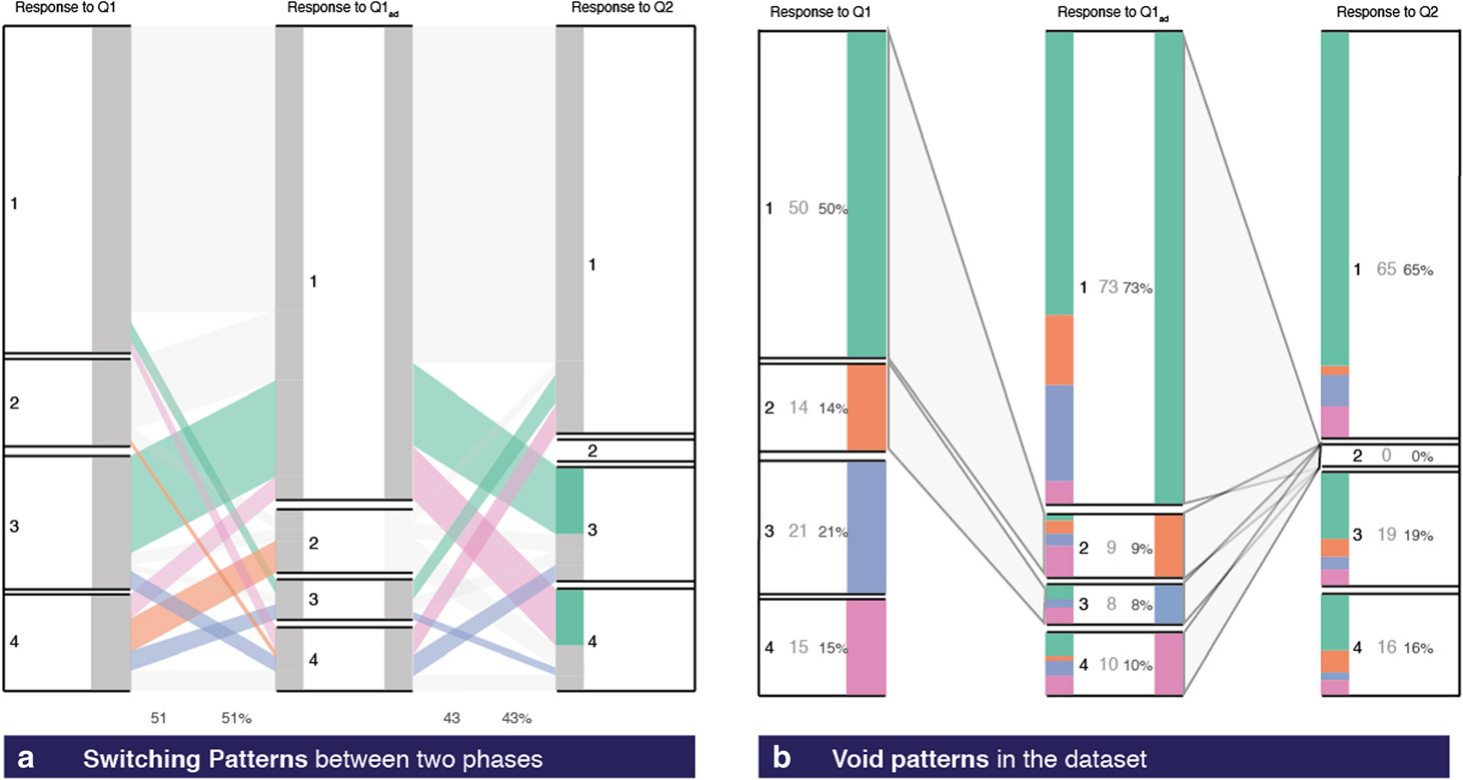
**a** Switching patterns. **b** Void patterns

Between two strata, a portion of one cohort can transit to the other across phases. A pair of such cohort represents a *switching pattern*. Figure 12a highlights the switching patterns in our dataset. While 3 % of students who gave an answer as option 3 changed their response to option 4, an equal percentage of students switched their response from option 4 to 3. A total of 51 % of the population migrate option choices in Q1 during two voting phase and 43 % for the second transition. Minimum 11 % of the population switch in pairs, for each first and second transition. *Switching* patterns between incorrect responses highlight that there exists a cohort who changes their responses across two phases but still remains incorrect. A higher proportion of them might indicate higher engagement but not necessarily improved learning. The instructor can evaluate which cohort of switching is most prominent and decide on some activities for clarifying both the groups.

#### Void

When no transitions take place between any two strata, they form the *void pattern*. For example, there is no cohort that migrates from a correct answer to choose the incorrect option 2 during re-voting (see Fig. 12b). Similarly, none of the students change from option 2 to 3. *Void* transitions between a correct to incorrect response are desirable. If there is *void* in an incorrect category, then it indicates that such alternate conceptions no longer exist after the intervention. In our regenerated dataset, proportion of option 2 as response in Q1_ad_ decreases with respect to proportion in response to Q1. In response to Q2, none of the learners choose that. Linking this to alternate conception, the pattern highlights the possibility that the PI activity helped in conceptual understanding of the student to eliminate the specific wrong approach in option 2 totally over the isomorphic question activities.

Table 1 summarizes the various patterns that emerge out of the transitions of the three-phase iSAT visualization.

**Table 1:** Patterns of transitions in iSAT

Patterns	Description
Aligned	Cohort that remains in the same stratum across phases
Starburst	Cohort that transits from a particular stratum in a pre-phase to other more desirable strata in the post-phase
Slide	Cohort that transits from a desirable stratum in the pre-phase to a less favorable stratum in the post-phase
Returns^a^	Cohort that is in an initial stratum in first phase but transits to a different category in the second phase and later returns back to the original stratum
Switching	Pair of cohort that represent a switch between initial categories across consecutive phases
Attractor	Cohort that migrates into a particular stratum in a post-phase from other strata in the pre-phase
Void	When no transitions take place between any two strata

^a^Requires at least three phases

## More uses of iSAT for instructors

Technology enables gathering of data towards informed teaching-learning practices (Vickrey et al. 2015). Often, learning dashboards visualize and make data accessible for various stakeholders in education. In a review study analyzing 24 dashboards, Verbert et al. (2014) suggest that there are three contexts in which these dashboards are utilized. These contexts are traditional face-to-face lectures, group work or classroom orchestration, and blended and online learning. Typically, data analyzed are artifacts produced by learners, social interactions among them, resources used, time spent, and various assessment results. But 23 out of the 24 dashboards essentially process data from captive logs of the application that they are associated with and do not allow users to upload, visualize, or interact with their own dataset independently. iSAT allows users to upload their own dataset locally on their own browser and analyze transition patterns across their attributes to gain insights.

We described the intricate patterns that emerge if we visualize the clicker responses of the example PI activity with isomorphic question. Face-to-face instructors can use iSAT to analyze interesting transitions in students’ attribute values during a classroom activity or across their course. For example, apart from performance patterns across a period, they can analyze temporal variation in students’ quantified motivation, engagement, etc. It can draw their attention to whether the consistently low performers are above the concerned threshold proportion in the class. Thus, iSAT can help visualize and analyze patterns of temporal variation of the same attribute.

iSAT can also visualize transitions across attributes like performance and perception. Stratifying performance level for instance as *high*, *medium*, and *low* and perception as *agree*, *neutral*, and *disagree*, iSAT can then help to highlight patterns like what percentage of high performers agree to a certain perception in a given student survey. Based on such transition patterns, the instructor can estimate effectiveness of their instructions.

Similarly, instructors who are using online medium can analyze different data that is logged in their system. Instructors can possibly find interesting patterns while visualizing transitions between resource use and active engagement or performance. The combination of the attributes chosen to trace the transitions can help the instructor with specific instructional decision-making. Note that there are cases, for example, visualizing transitions between perception and performance, one should not interpret causality from this visualization. In the next section, we explain the workflow of deciding the phases and strata of iSAT from a given dataset that instructor wants to visualize and interactively analyze.

### How can instructors use iSAT

**Fig. 13:**
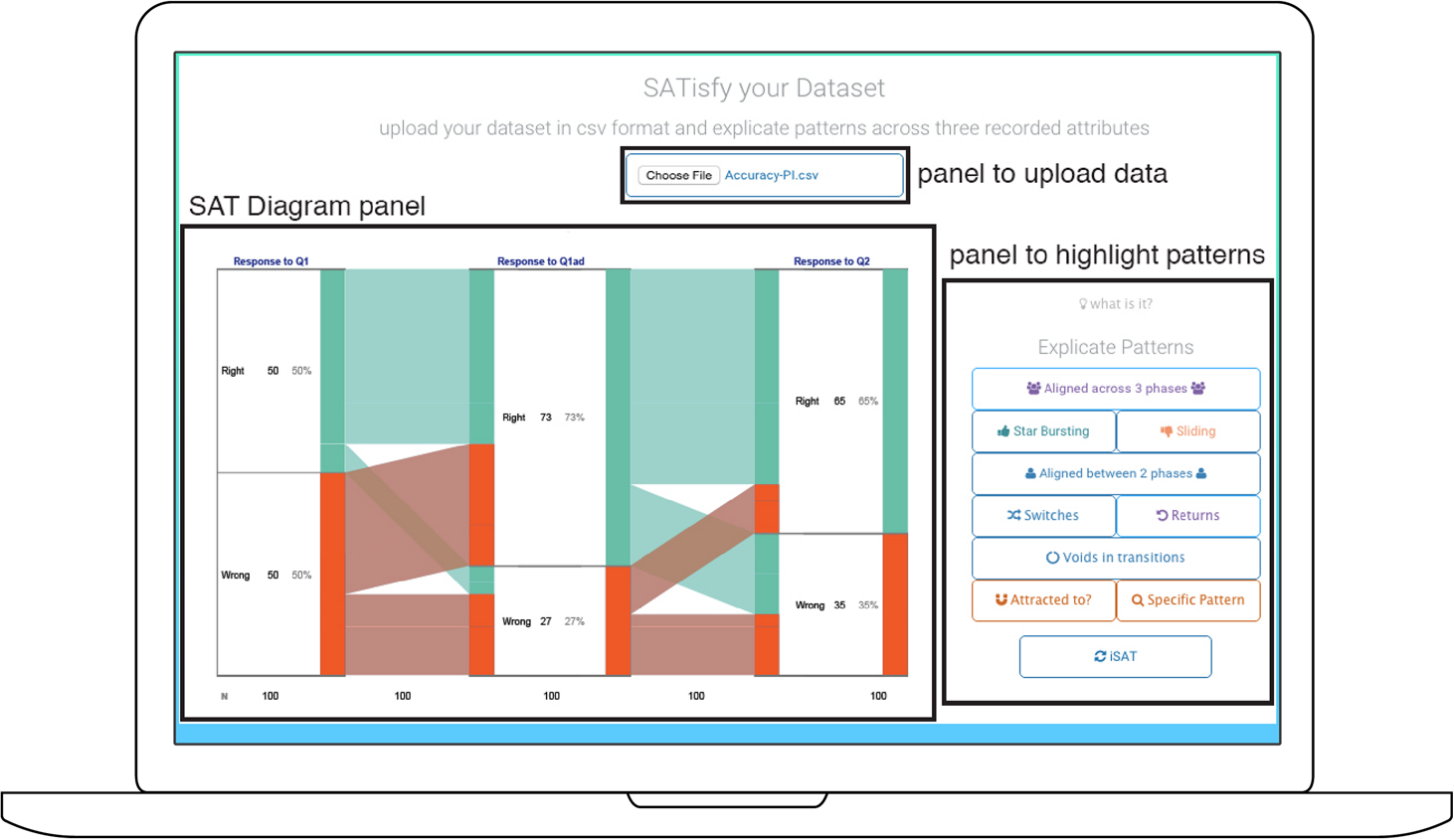
UI of the iSAT tool

The iSAT portal is accessible at www.et.iitb.ac.in/iSAT. The tool is accessible from the *SATisfy your Data* button. The desktop UI of the tool is presented in Fig. 13.

**Fig. 14:**
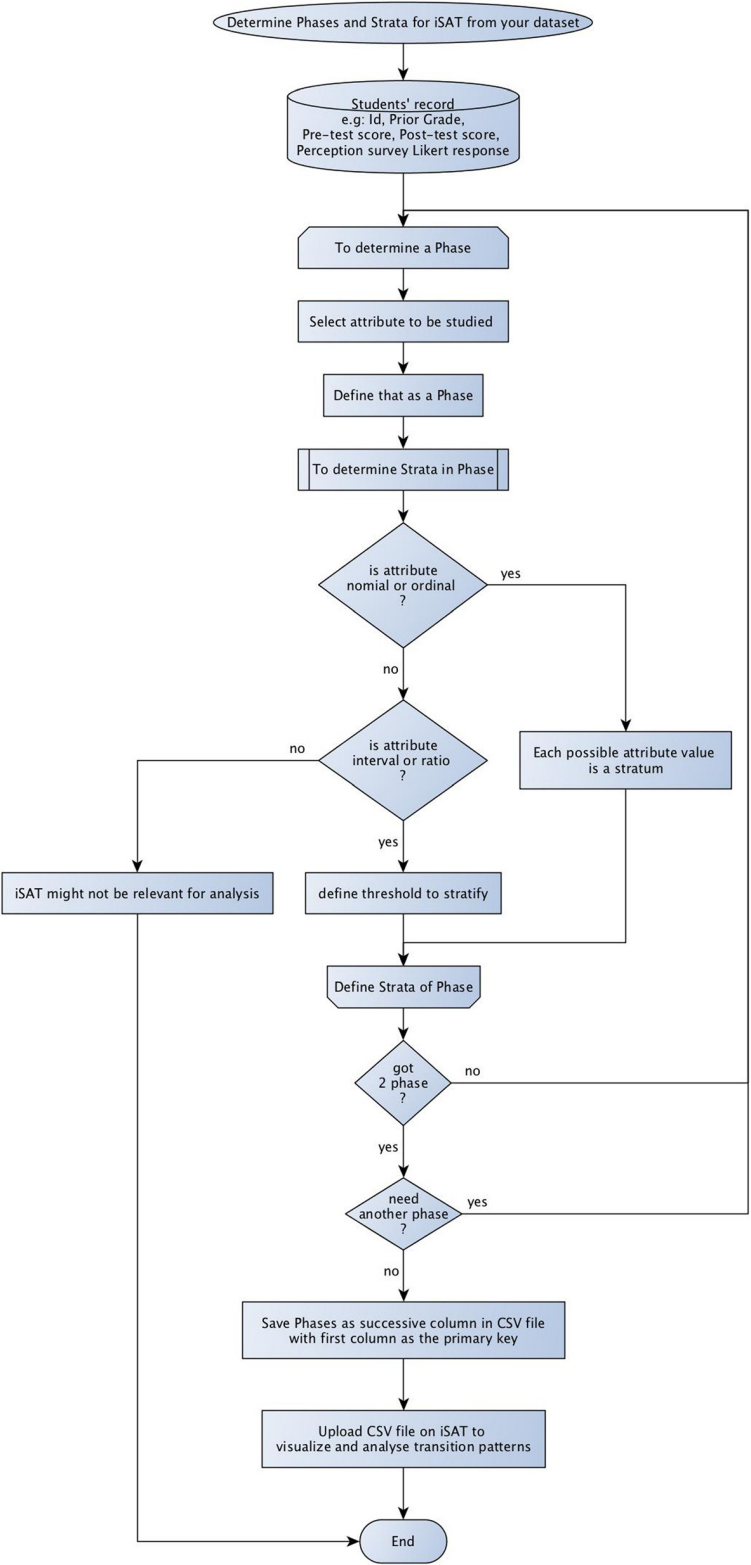
The workflow to determine the phases and strata of iSAT from the collected dataset

Instructors can follow the step-by-step workflow shown in Fig. 14 to determine the phases and corresponding strata of any possible iSAT visualization in their dataset. Considering the PI dataset, which has records of the form {Student ID, Response to Q1, Response to Q1ad, Response to Q2}. *Response to Q*_*1*_ is an attribute that the instructor chooses as the first phase. The attribute stores the response option of each student (1/2/3/4), which is a nominal variable. Hence, all the possible attribute values (1/2/3/4) are represented as strata. This completes the determination of the first phase (*response to Q1*) having four strata {‘1’, ‘2’, ‘3’, ‘4’}. As minimum two phases are required to visualize a transition, the flow directs to choose another phase. The instructor then chooses the response after discussion (*response to Q1*_*ad*_). Similar to previous cycle at the end of this cycle, there are two phases each having four strata. In the next cycle, the instructor chooses *response to Q2*. Then, the users need to save the data of each determined phases as consecutive columns in a CSV file with the first column as a primary key. The column heading is taken as the label for the phase. For example, a valid CSV file to generate Fig. 5 would have the header row as {Student ID, Response to Q1, Response to Q1_ad_} and a sample record as {46, Wrong, Right}.

### Earlier research use of iSAT

In earlier works, mainly researchers used the SAT diagram to trace transitions in dataset. One study defined a model of engagement during different phases of Think-Pair-Share (TPS), an active learning strategy, using the SAT diagram (Kothiyal et al. 2013). The diagram also traced transition in pre-test and post-test performances in the two-group study that evaluated effectiveness of TPS in a large undergraduate CS101 course (Fig. 1 visualizes the transition pattern for the experimental group. It is used as an example in this paper) (Kothiyal et al. 2014). Other research studies used iSAT visualization to trace transition patterns across multiple attributes of learners. Mishra and Iyer (2013) traced students’ performance across paper tests and problem posing activity. Warriem et al. (2013) tracked online learners’ performance in participation for completing and submission of assessment. Majumdar and Iyer (2014) studied consistency of survey responses across different questions asked to understand student’s perception, using iSAT. Readers who are interested in details of evolution of iSAT across its design iteration can refer to Majumdar et al. (2014).

## Workshops to introduce iSAT to instructors

We conducted two hands-on workshops to introduce the iSAT tool to the instructors’ community. In this section, we discuss the context of the workshop and then report a study done as a part of it. We could validate the steps developed to visualize transitions and its method of analyzing educational dataset. The preliminary study indicated that instructors, who were participants in those workshops, perceived the tool was an acceptable system relevant to their job.

### iSAT training workshops

Out of the two workshops, the first one was a part of the Mentoring Educators in Educational Technology (MEET) 2015 workshop (Warriem, 2015). The second opportunity was at IEEE Technology for Education (T4E) conference, where it was accepted as a workshop (Majumdar and Warriem 2015). During T4E, the same training session was repeated twice to accommodate willing participants. All the sessions were 1.5 h in duration.

#### Workshop objective

The overall objective of both the workshops was to introduce iSAT as a tool for analyzing the data that instructors deal with in their classroom activities. It was in alignment with the MEET workshop objective to facilitate engineering college instructors to reflect on their instructional practices by conducting classroom action research. The iSAT session there focused on analyzing cohorts in the classroom. During T4E, the objective was to introduce iSAT as a visual learning analytics tool to trace educational dataset.

#### Participants

The first training session during the MEET workshop had 14 participants. Eleven of them were in-service engineering college instructors with minimum of 2 years of teaching experience. They were from Computer Science, Electrical Engineering, and Mechanical Engineering departments. Among the participants, two had a PhD degree, three were pursuing part-time doctoral program in their respective domains, and three were Masters students of Computer Science.

The training workshop during T4E had a total of 42 participants. In a pre-workshop demographics survey, 28 of them said they were college-level instructors.

#### Implementation

The workshop introduced iSAT with examples using data collected in the context of several/various active learning interventions. Peer instruction (PI) was selected as one such context, and patterns in user response transitions during PI were used to demonstrate iSAT. Then, limitations of multiple histograms and usefulness of iSAT were highlighted through series of questioning activities given to the participants. Then, the participants used the iSAT tool with sample data from prior study (Kothiyal et al. 2014). It consisted of pre-test and post-test performance data of two groups of learners. One group participated in a TPS activity versus others had regular lecture-based instruction in a CS101 course.

#### Example of participant tasks

We designed a worksheet for the participants that had multiple tasks to analyze transitions in the TPS performance data using iSAT. While answering the queries posed in each task, the participants utilized the features of iSAT and interpreted the data at four different levels. Table 2 gives the four levels of task and their corresponding examples.

**Table 2:** Levels of task and their examples

Levels of task	Example
Identify characteristics of the phase	What percentage of students from the control group performed low in the post-test? What is the ratio of low performers to high performers for the post-test in the experimental group?
Identify characteristics of the transitions	How many low-scorers in the pre-test within the control group improved to high scores in the post-test? Is this statement about the experimental group true: “More than half of the high scorers in the pre-test scored high even at the end of the post-test”?
Identify patterns in the transitions	What is the percentage of learners showing the same level of performance in the pre-post-tests within the control group? Find the ratio of the number of learners whose performance improved to those whose performance deteriorate across the pre- and post-tests within the experimental group.
Compare two SAT diagrams	What is the ratio between transitions from low-high in the control group to the same in the experimental group? Compare and contrast effects of TPS on high achievers in the control and experimental groups.

### Preliminary usability and usefulness study with instructors

We carried out a research study to evaluate the usefulness and usability of iSAT. Our research questions, methodology, and analysis technique are discussed in this section.

#### Research questions

The workshop introduced a method of cohort analysis with educational data that instructors collect in their teaching-learning environment. The iSAT tool has multiple functionalities, which assists the user from uploading their dataset to exploring transition patterns interactively. Hence, we wanted to separately study the usefulness and the usability of the tool.

Our first research question (RQ1) is as follows: *How useful is iSAT as perceived by the instructors?* We studied the usefulness of the tool with respect to the intention of using the tool by the workshop participants, its perceived usefulness after the introductory session, and the relevance of such analysis in their job.

Our second RQ2 is as follows: *How usable is iSAT as perceived by the instructors?* Given the multiple functionality of the tool, we studied how useful was the tool for the workshop participants.

#### Instruments for data collection

We have used survey questionnaires as the instrument to evaluate the usefulness and usability of iSAT. To answer RQ1, we had chosen constructs from the Technology Acceptability Model 2 (TAM2) (Venkatesh & Davis, 2000) and adopted eight items related to the intention to use (Q1 & 2), perceived usefulness (Q3–Q6), and job relevance (Q7–Q8). For RQ2, we have used SUS (Brooke, 1996) as our instrument. With each SUS item, we had requested to elaborate the reason for their response. We also received verbal feedback from certain participants after the workshop that expressed how iSAT was useful in their own context.

#### Sampling

The participants of the two workshops together are considered as the sample of the study. There were a total of 56 participants, who entered the demographics data. Among them, we received 30 SUS responses and 12 responses for the usefulness survey.

#### Analysis

The TAM2 items had 7-point Likert’s scale response, and the SUS survey asked 5-point Likert’s response. To answer RQ1, Likert’s scale data was aggregated in three groups, viz agree (combining responses 5 to 7), neutral (response 4), and disagree (combining responses 1 to 3). We further analyzed the transitions of response from one group to another across the survey items by using iSAT. This helped to explicate patterns across the three constructs chosen from the TAM2 survey. For RQ2, we considered the total SUS score and evaluated its usability with respect to empirical results of SUS score (Bangor et al. 2008). We examined the reason that participants gave corresponding to their response to each items to find the major perceived reason.

#### Results and interpretations

**Fig. 15:**
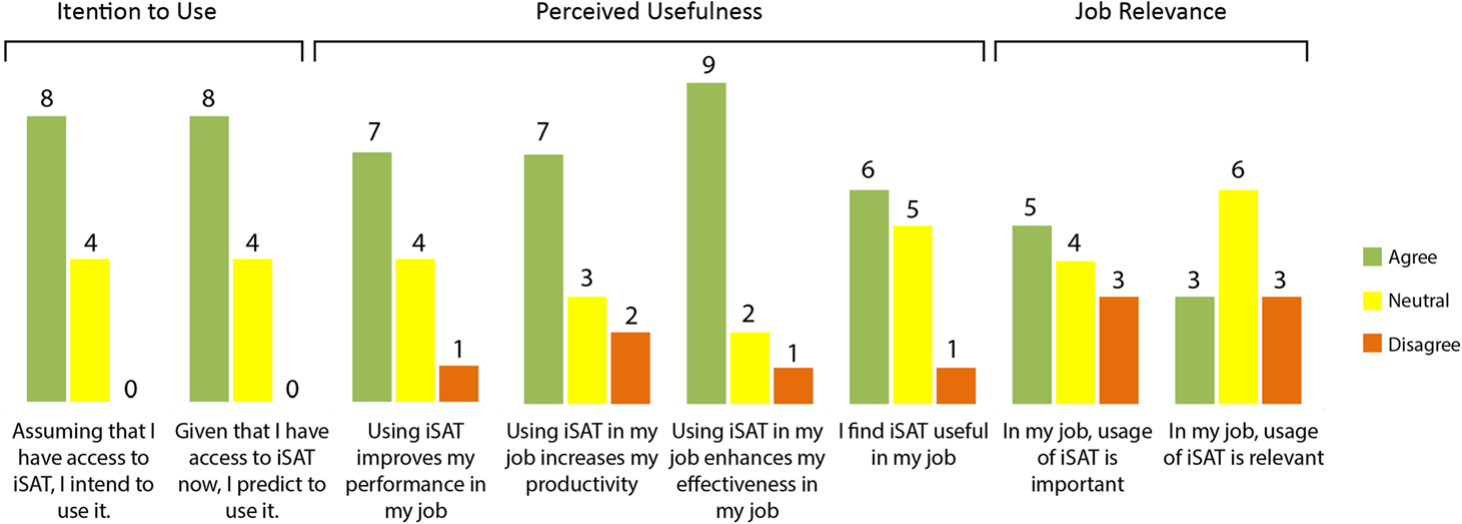
The distribution of responses of usefulness survey. (*n* = 12)

The distributions for the usefulness survey items are presented in Fig. 15.

**Fig. 16:**
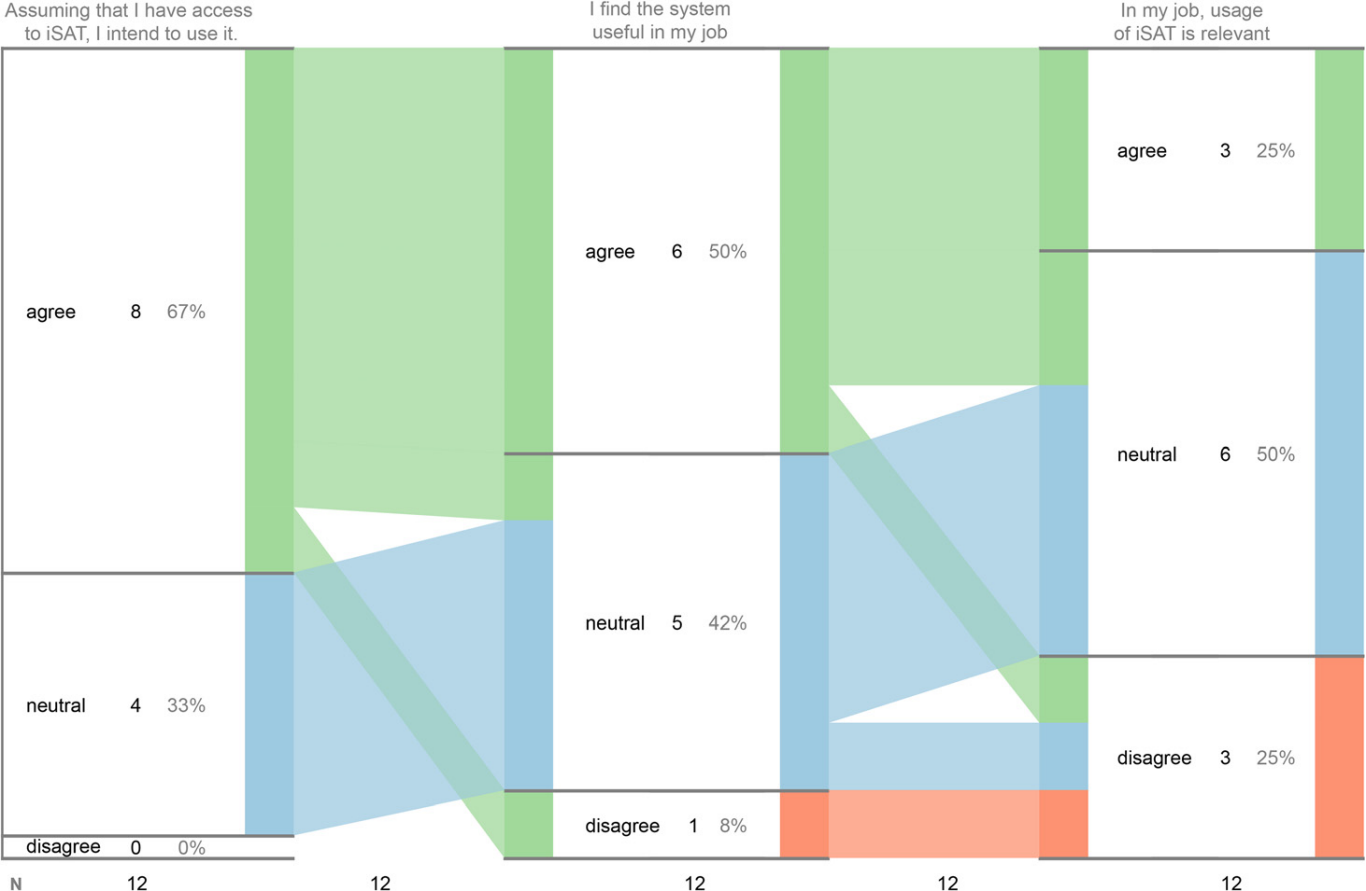
Transitions of response across the constructs of usefulness survey

Among the responses, eight (67 %) showed intent to use iSAT given a chance to access the tool assuming or given a chance to access iSAT. Fifty-eight percent of responses confirmed perceived increase in performance and productivity, whereas 75 % perceived enhanced effectiveness and 50 % found iSAT useful in their job. Forty-two percent perceived usage of iSAT was important in their job, and 25 % agreed it was relevant. Figure 16 shows the iSAT visualization of the transitions in the usefulness constructs.

Analyzing the transitions in survey response through iSAT, we further found three responses (25 %) were consistently agreeing across all the three constructs. Within the construct of intention of use, there were two items. Seven responses (58 % of total and 87.5 % of the responses which agreed in individual items) agreed in both the items, and two responses (16 %) switched options across these two items, one from being neutral to agreeing and the other vice versa. Three (25 %) were neutral for both the items. None of the responses disagreed about the intention of use. This established that participants intended to use iSAT. Only one response consistently disagreed to the perceived usefulness and job relevance of iSAT, though it also presented intention to use. Though the survey used validated instruments to elicit participants’ response, it has certain limitations. The survey was taken immediately after introducing iSAT in a 1.5-h workshop session. That might have an overwhelming effect, which resulted higher agreement in intention to use. Similarly, higher percentage of neutral response in the job relevance construct is because the participants wanted to explore the iSAT tool further before estimating the relevance.

The mean SUS score was 71.58 (s.d. 18.02, *n* = 30). The score reflects that iSAT is an acceptable system. The reasons noted by some users corresponding to each item in the SUS survey confirmed why iSAT was perceived usable and highlighted some of the issues that the user faced. The participants mentioned “the tool (iSAT) can be used for other type of analysis than Statistical analysis,” “its easy to understand the GUI,” “no need of an expert to learn to use the tool, because as you start exploring the tool, the transformation in the pattern itself gives an idea of the cohort,” “could use the functions efficiently which gave clear results,” and “results seems fairly correct.” Other feedback were “without using for couple of times we cannot answer it (whether cumbersome to use),” “practice will make it user friendly,” “(cumbersome to use) may depend on how many parameters,” “(I felt confident) because it is shown how to use,” “few things which anyone can get through,” and “It is not complex, but without a guide it might be little difficult to figure out certain functions.” All of these feedback statements gave us inputs on what is required to emphasize for the next set of workshops and possible modifications in the iSAT tool itself to support them more regarding organizing the data.

#### Usage of iSAT by workshop participants

There were two instances where workshop participants used iSAT after attending the session and reported findings in international conferences, though this usage does not intend for real-time instructional decision-making. But both the instructors mentioned that analyzing their data with iSAT helped them to investigate and demonstrate the dynamics of their class.

One participant from the MEET workshop later collaborated with her colleague and conducted a project-based learning (PBL) activity in an automobile engineering course across a semester, in her college. They investigated the transition of students’ performance while they participated in the PBL activity. The instructors designed assessments of higher order thinking skills (HOTs) and lower order thinking skills (LOTs). The phases for iSAT were the different assessment topics in LOTs (recall and apply) and HOTs (analyze, evaluate, and create). They defined high-medium-low performance level and traced the transition patterns of students across the assessments, separately for the two levels of thinking skills. Further, cumulating the score for LOTs and HOTs, they could analyze transitions between the two levels of thinking skills. Mistry et al. (2016) reported their findings in Learning and Teaching in Computing and Engineering (LaTiCE 2016).

Another participant who attended the T4E workshop used the tool the very next day to present her data during the paper session. Later, we had corresponded with her on mail to gather details of how she utilized iSAT. To quote her, “I have used the tool for analyzing the POGIL INDIA data that I have collected from my class rooms. … this tool (iSAT) made a way for me to show my data in a proper form.”

## Conclusion

Earlier studies on iSAT have demonstrated its development process over three research-based design iterations (Majumdar et al. 2014) and its potential to be used for learning analytics (Majumdar and Iyer 2014). In this paper, we describe the utility of iSAT for instructors. Our iSAT tool enables an instructor to explicate transition patterns across attributes in their dataset at an appropriate level of granularity and explore them interactively in real time. To illustrate with an example context, we choose an extended version of PI that has an isomorphic second question. The transitions observed were across the voted options in the three phases of voting (Q1, Q1_ad_, Q2). Well-designed PI questions link alternate conceptions to the wrong options. iSAT can then help the instructors to visualize such transitions of the alternate conceptions as evident from their students’ response data. We identified seven possible patterns that are relevant to analyze the three-phase example activity. Given different contexts, those patterns can be interpreted accordingly. Identifying the cohorts from the transitions and its patterns can assist the instructor to estimate the trend and take informed decisions.

The workflow of using iSAT with dataset (see Fig. 14) gives a step-by-step procedure to determine what are the phases and corresponding strata that can be visualized. Depending on the specific attributes that the instructor wants to visualize, one can get informed about temporal trends (e.g., variation of performance across assessments), trends across different attributes (e.g., transitions across performance and perceptions), or even a mixed analysis (e.g., transitions across the performance across pre-post-tests and perception data).

The iSAT introductory workshops for the instructors helped us to gather their perception regarding the usefulness and the usability of iSAT. While the MEET participants agreed iSAT was an interesting tool to adopt in one’s teaching-learning environment, they also emphasized that more exposure to the tool and its context is required before they could integrate in their practice. Based on their feedback, we added an active example of PI during the T4E workshop. The participants had to respond to the PI questions in phases. Then, their collected response dataset itself was used to demonstrate the iSAT tool. This helped in having a current context, which assisted them in interpreting the transition patterns. Also, the four levels of queries given in the worksheet were structured during the second offering. The survey results indicate the usefulness and usability aspect of the iSAT tool as perceived by the participating classroom instructors.

We are still improving iSAT based on the feedback received from the workshop participants. We plan to extend the interactive visualization for more than three phases. But we have seen that the transition patterns often become complex and difficult to analyze. Hence, we are working to add a pre-module to assist the users to decide the required phases and strata based on the attributes of their dataset. A set of guided questions would be presented to the users, at the end of answering which they can decide what are the transitions they want to trace. We believe having clarity of what transitions the users want to trace would also help them to interpret those transitions in the context of the data collected. Then, choosing appropriate three phases for analysis can also give insights to the context. Though the participants’ perception survey indicates the preliminary usefulness and usability, once instructors adopt and utilize iSAT for in-class instructional decision-making, further user study can be conducted to investigate the mechanism of how iSAT actually assists them. We are currently examining utility of iSAT to analyze data gathered from a MOOC setting. Having MOOC participants’ data regarding their engagement, performance and perception, iSAT can highlight the patterns of transition across these attributes. It shall help the various stakeholders of MOOC to understand the dynamics of the cohorts in the offered course. MOOC instructors can possibly decide specific intervention for certain cohorts and further compare patterns across offerings of the same course to determine its effectiveness.
